# Oxidative Stress Biomarkers, Nut-Related Antioxidants, and Cardiovascular Disease

**DOI:** 10.3390/nu12030682

**Published:** 2020-03-03

**Authors:** Julia Lorenzon dos Santos, Alexandre Schaan de Quadros, Camila Weschenfelder, Silvia Bueno Garofallo, Aline Marcadenti

**Affiliations:** 1 Graduate Program in Health Sciences (Cardiology), Institute of Cardiology of Rio Grande do Sul/University Foundation of Cardiology (IC/FUC), Princesa Isabel Avenue, 395, Porto Alegre, 90040-371 Rio Grande do Sul, Brazil; julia.lorenzon@gmail.com (J.L.d.S.); consult.asq@gmail.com (A.S.d.Q.); camilawesche@gmail.com (C.W.); silviagarofallo@hotmail.com (S.B.G.); 2 HCor Research Institute, Coracao Hospital (IP-HCor), Abílio Soares Street, 250, 04004-05 São Paulo, Brazil

**Keywords:** oxidative stress, antioxidants, nuts, cardiovascular diseases

## Abstract

Atherosclerosis is related to fat accumulation in the arterial walls and vascular stiffening, and results in acute coronary syndrome which is commonly associated with acute myocardial infarction. Oxidative stress participates in the pathogenesis of atherosclerosis. Thus, the inclusion of food sources of dietary antioxidants, such as different kinds of nuts, may improve biomarkers related to oxidative stress, contributing to a possible reduction in atherosclerosis progression. This article has briefly highlighted the interaction between oxidative stress, atherosclerosis, and cardiovascular disease, in addition to the effect of the consumption of different nuts and related dietary antioxidants—like polyphenols and vitamin E—on biomarkers of oxidative stress in primary and secondary cardiovascular prevention. Studies in vitro suggest that nuts may exert antioxidant effects by DNA repair mechanisms, lipid peroxidation prevention, modulation of the signaling pathways, and inhibition of the MAPK pathways through the suppression of NF-κB and activation of the Nrf2 pathways. Studies conducted in animal models showed the ability of dietary nuts in improving biomarkers of oxidative stress, such as oxLDL and GPx. However, clinical trials in humans have not been conclusive, especially with regards to the secondary prevention of cardiovascular disease.

## 1. Introduction

Atherosclerosis, succinctly defined as arterial degeneration associated with increased fat accumulation in the arterial walls and an increased vascular stiffness [1], can result in acute coronary syndrome (ACS), a severe cardiac disorder that is commonly associated with acute myocardial infarction (AMI) [2,3]. AMI is the leading cause of fatality worldwide, responsible for more than nine million deaths yearly [4]. Oxidative stress participates in the pathogenesis of various diseases [5], including those of atherosclerotic origin. Damage caused to the deoxyribonucleic acid (DNA), ribonucleic acid (RNA), proteins, lipids, and the plasma membrane of the cell or the inner mitochondrial membrane and the nuclear envelope, are all implicated in an impaired circulatory and cardiac function [6,7]. In this sense, strategies that decrease the production of reactive oxygen species (ROS) should be used in the prevention and treatment of cardiovascular disease (CVD), including atherosclerosis and ACS [2,8].

Nutrients show antioxidant activities, especially vitamin E and phenolic compounds [9], with nuts standing out as a source of both [10]. In vitro studies [11,12,13,14,15,16,17,18], animal models [19,20,21,22,23,24], observational studies [25], and randomized trials [26,27,28,29,30,31,32] suggest the potential benefit of including different nuts in the diet to improve biomarkers of oxidative stress.

This article has briefly discussed the role of oxidative stress in the genesis of atherosclerosis and ACS, and has assessed studies that have evaluated the effect of dietary nut supplementation on oxidative stress parameters in the primary and secondary prevention of cardiovascular diseases. 

## 2. The Role of Oxidative Stress in the Development of Atherosclerosis and ACS

Oxidative stress participates in the pathogenesis of atherosclerosis and its risk factors (namely, hypercholesterolemia, hypertension, and smoking) and increases free radical production in the vascular wall [33].

The resulting accumulation of DNA damage is associated with aging and age-related diseases, as it promotes apoptosis and cell senescence [33,34]. In addition, the vascular smooth muscle cells (VSMC), which play an important role in atherosclerosis development, are activated by DNA damage and replication and secrete high levels of senescence-associated secretory phenotype (SASP) factors with inflammatory characteristics, capable of promoting pro-atherosclerotic conditions [34,35] in the adjacent cells.

**Figure 1:**
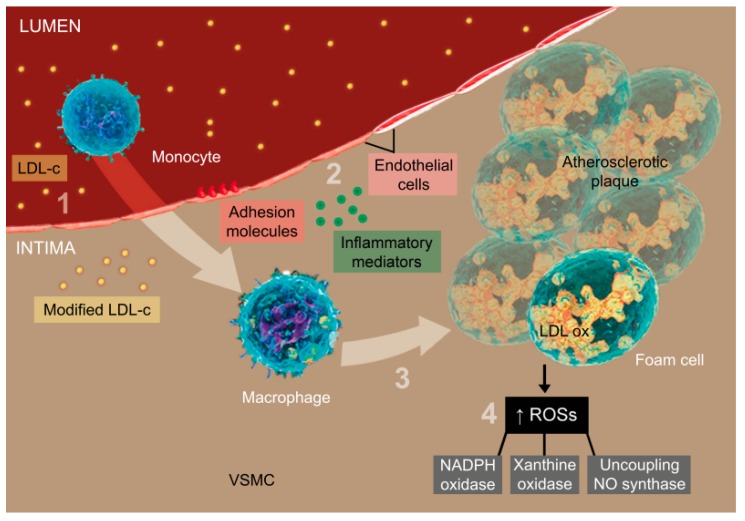
Representation of the atherosclerotic process adapted from Glaudemans et al. [40]. (1) Migration of LDL-c to the endothelial wall and its modification; (2) Uptake of the circulating monocytes by endothelial cells and VSMCs; (3) Transformation of monocytes into foamy macrophages from the uptake of LDL-c by scavenger receptors; (4) Increase of ROS and oxidation of LDL-c. Abbreviations: LDL-c: Low-density lipoprotein cholesterol; LDL ox: Oxidized LDL-c; VSMC: Vascular smooth muscle cells; ROS: Reactive oxygen species; NO: Nitric oxide.

Hypercholesterolemia is a major risk factor for atherosclerosis. An increase in the plasma cholesterol levels results in an endothelial dysfunction that facilitates the migration of lipids, especially that of the low-density lipoprotein cholesterol (LDL-c), into the arterial wall, where it is modified by ROS molecules, such as NADPH oxidase, xanthine oxidase, enzymes of the mitochondrial respiratory chain, and by the decoupling of the endothelial nitric oxide (NO) synthase. Endothelial cells and VSMC express adhesion molecules and chemotactically recruit the circulating monocytes which migrate to the subendothelial space where they are transformed into macrophages and then foamy macrophages by an LDL-c oxidization; the degree of oxidation correlates with the severity of the disease. This process results in a cascade of vascular changes that have clinical sequelae, including the narrowing of vessels, causing associated clinical symptoms (angina pectoris), and ACS of different types based on the stability of the atherosclerotic plaque [36,37,38,39]. ACS is a subcategory of coronary artery disease (CAD), which has characteristic symptoms and is often associated with AMI [2] (Figure 1).

In the context of atherosclerosis, some oxidative stress biomarkers and endogenous antioxidants have great clinical relevance, including the oxidized LDL-c (oxLDL), plasma total antioxidant capacity (TAC), superoxide dismutase (SOD), catalase, and glutathione (GHS). In addition, foods rich in exogenous antioxidants are closely related to having cardiovascular protective abilities. 

## 3. Nuts and Their Antioxidant Properties

Nuts (tree nuts) and peanuts are rich sources of antioxidants, particularly vitamin E and polyphenols. In addition, other characteristics of these foods support recommending their dietary inclusion in CVD prevention and treatment.

### Definition and Nutritional Composition of Different Nuts

Nuts are dry, thick fruits with thorny-covered seeds [41]. The best known are almonds, hazelnuts, Brazil nuts, cashews, macadamias, walnuts, pecans, and pistachios. Peanuts and Baru almonds are edible seeds classified as legumes, not nuts since their grains are produced in pods [41,42]. However, the characteristics and nutritional composition of peanuts are similar to those of nuts and they are considered as oleaginous fruits.

Nuts are composed of macronutrients [43,44], containing high levels of proteins and unsaturated fats, in addition to dietary fiber [44], micronutrients [44], fat-soluble bioactives [44,45] which include polyunsaturated fatty acids (PUFA) and monounsaturated fatty acids (MUFA), and phytochemicals [46], such as phenolic compounds. These nutrients, in synergy, appear to be responsible for the beneficial effects of nuts on human health [46,47,48,49,50,51,52].

The lipid content of nuts, peanuts, and Baru almonds varies between 30%–60% of their approximate composition [52,53]. Regarding the fatty acid profile of these foods, high levels of oleic (C18:1) and linoleic (C18:2) [53] fatty acids are notable. Table 1 presents the fatty acid profile of the main nuts and edible seeds.

Phytochemicals are considered non-nutrient antioxidants [54] present in high concentrations in nuts which are considered a major dietary source of these antioxidants [55]. Phytochemicals present in nuts have bioactive properties, such as antioxidant, antiproliferative, anti-inflammatory, antiviral, and hypocholesterolemic properties [56]. However, the phytochemical content can vary considerably according to nut type, genotype, pre- and post-harvest conditions, and storage conditions [46]. Table 2 shows the phytochemical content of nuts and edible seeds.

Edible nuts and seeds are also sources of nutrient antioxidants, such as selenium, zinc, and vitamins A, C, E, with emphasis on tocopherols [54,57,58] (Table 3). However, their metabolic effects seem to be dependent on the bioaccessibility in the gastrointestinal tract, which may be influenced by dietary fiber and MUFA content of the nut [59].

## 4. Nuts, Antioxidants and Oxidative Stress: Studies In Vitro and in Animal Models

### 4.1. Studies In Vitro

An in vitro study with plasma and platelets incubated in proanthocyanidin fraction from Medemia argun nuts (0.5 μg/mL–50 μg/mL), demonstrated a decrease in oxidation of the thiol and the carbonyl groups, and a significant increase in GSH, compared to that observed with peroxynitrite (ONOO^-^) treatment only [11]. These polyphenols exert their antioxidant activity by DNA repair, lipid peroxidation prevention, modulation of the signaling pathways, and inhibition of the mitogen-activated protein kinases (MAPK) pathways through the suppression of nuclear factor kappa B (NF-κB) and activation of the nuclear factor erythroid 2-related factor 2 (Nrf2) pathways [12]. Oocytes of secondary follicles from lambs maintained for 18 days in control medium containing polyphenol resveratrol (2 μM, 10 μM, or 30 μM) exhibited significant decreases in the intracellular GSH levels at 10 μM and 30 μM concentrations compared to 2 μM due to a decreased mitochondrial activity [13].

Different nuts (almonds, macadamias, hazelnuts, pistachios, and walnuts) showed that their supernatant fractions displayed antioxidant effects after being fermented in vitro [14]. An investigation into the antioxidant effect of pistachios in the macrophage/monocyte cell line, J774, found that pretreatment with pistachio shells or roasted and salted pistachios in different concentrations decreased the production of ROS and malondialdehyde (MDA) significantly (*p* < 0.01) [15].

Walnut oil, in different concentrations (40 μL/mL–319 μL/mL) in U937 cells cultured for 24 h to 72 h, had positive effects on SOD activity, with the greatest induction (*p* ≤ 0.01) seen within 48 h of incubation [16]. Additionally, defatted walnut flour exerted good antioxidant activity on the peroxide-injured nerve cells through the elimination of the hydroxyl radical and reduction of ROS in the selected peptides [17].

The effects of walnut extracts rich in polyphenols on human plasma in vitro was compared to ellagic acid, with a reduction in LDL-c oxidation of 87% and 38% observed, respectively; the copper-mediated LDL-c oxidation was inhibited by 84% in the presence of the extract (compared to 14% by ellagic acid) [18].

### 4.2. Studies in Animal Models

Male Wistar rats were divided into six groups [n: six per group: (1) control, (2) 75 mg of Brazil nut (BN), (3) 150 mg BN, (4) ischemia-reperfusion (IR), (5) IR with 75 mg BN, and (6) IR with 150 mg BN and submitted to BN daily seven days prior to surgery for IR, with animals sacrificed 48 h thereafter. Reduced oxidative stress—including elevated expression of inducible nitric oxide synthase (iNOS), nitrotyrosine renal expression, and plasma thiobarbituric acid reactive substances (TBARS), was observed in mice pretreated with BN (similar results with both doses) [19]. Male mice C57BL/6J fed either a common feed or fat-rich diet (45% or 21.5% energy from walnuts) for six weeks showed a decrease in MAPK changes and a reduction in lipid peroxidation [20]. Male mice fed with an atherogenic diet supplemented with mixed nuts (almonds, macadamias, peanuts, pistachios, cashews, Brazil nuts, and walnuts) or isolated pistachio showed significantly decreased concentrations of oxLDL as compared to that observed in the control knock-out mice (*p* = 0.044) [21]. Furthermore, apoE-deficient mice receiving supplementation (3%) of mixed nuts (almonds, hazelnuts, and walnuts) showed significantly decreased levels of oxLDL (*p* < 0.05) compared to the group receiving an isocaloric diet supplemented with palm oil [22].

Male rats fed with a high-fat diet supplemented with pecan oil, polyphenol extract, or part or whole pieces of nuts for nine weeks showed a greater increase in antioxidant enzyme activity (*p* < 0.05), especially in the group supplemented with pecan oil [23]. Hyperlipidemic rats fed a fat-rich diet and pistachios for eight weeks showed a reduction of TBARS measured MDA (*p* < 0.01), and, although not significant, an increase in SOD compared to that observed in the control [24].

## 5. Nuts and Oxidative Stress Biomarkers: Studies in Primary Cardiovascular Prevention

The current consensus indicates that the inclusion of nuts in the diet helps in the prevention of primary CVD [64,65]. A review of major epidemiological studies, such as the Adventist Health Study [66], the Iowa Women’s Health Study [67], the Nurses’ Health Study [68], and the Physicians’ Health Study [69] showed a 37% lower risk for CVD in individuals who consumed nuts more than four times a week compared to those who rarely or never consumed nuts; moreover, a reduction in risk of 8.3% was assigned to each weekly portion of nuts [25].

The *PREvención con DIeta MEDiterránea* (PREDIMED) study [64] conducted in Spain enrolled 7447 individuals at high risk for CVD, who participated in one of three dietary interventions: The Mediterranean diet (MeDiet) supplemented with an extra-virgin olive oil (MeDiet + EVOO), the MeDiet supplemented with mixed nuts (MeDiet + MN), and the control diet (low-fat standard as per the American Heart Association [AHA]). After a median follow-up of 4.8 years, there was a 30% lower risk observed for AMI, stroke, and CVD-related mortality in the group allocated with the diet supplemented with extra-virgin olive oil, while there was a 28% reduced risk in the group supplemented with mixed nuts, compared to that in the control diet.

Subanalysis [70] of the PREDIMED study evaluated biomarkers related to oxidative stress, identifying greater SOD and catalase plasma activity (*p* < 0.003 and *p* < 0.004, respectively) and less plasma xanthine oxidase activity (*p* = 0.008) in individuals undergoing interventions (the MeDiet + EVOO and the MeDiet + MN). Another subanalysis [71] published by the same authors, detected an improvement in the plasma non-enzymatic antioxidant capacity (NEAC) levels after one year of intervention through increasing plasma levels of potential iron-reducing antioxidant (FRAP) in both interventions [the MeDiet + EVOO: 72.0 µmol/L (95% CI, 34.2–109.9), and the MeDiet + MN: 48.9 µmol/L (24.3–73.5)].

Specifically regarding nuts, a study that evaluated the acute effect of consumption of four different forms of walnut [whole walnut (85 g), walnut oil (51 g), defatted walnut pulp (34 g), and walnut skin (5.6 g)] in overweight or obese individuals with moderate hypercholesterolemia, identified an increase in the antioxidant marker FRAP at all meals (*p* < 0.01), but this was less significant for defatted walnut pulp [72]. The nutritional intervention of one unit of Brazil nuts per day for three months in hemodialysis patients resulted in improved plasma glutathione peroxidase (GPx) levels and a reduction in the 8-hydroxy-2′-deoxyguanosine (8-OHdG) and in the 8-isoprostane levels (*p* < 0.001) [73].

A placebo-controlled, parallel randomized clinical trial (RCT) [26] of 46 overweight and obese women, divided into two groups (normocaloric and isoenergetic diet + placebo [PLA] or normocaloric and isoenergetic diet + 20 g of Baru nuts [BARU]), found that after eight weeks of intervention, the BARU group showed a significant increase in the GPx activity in comparison to the PLA group (+0.08 U/mg, 95% CI 0.05–0.12; *vs*. −0.07 U/mg, 95% CI −0.12 to −0.03, *p* < 0.01) accompanied by an increase in the plasma copper concentration (*p* = 0.037). However, no differences were observed in CAT, SOD activity or MDA concentration between groups. A further study [74] involving Baru nuts was divided into two periods of six weeks (four weeks of washing between periods) and conducted in 20 slightly hypercholesterolemic individuals. Participants were instructed to follow the supplementary diet of 20 g/day of Baru nuts or placebo, and at the end of the study, no changes were observed in the biomarkers of oxidative stress investigated.

Three RCTs were conducted involving dietary intervention with Brazil nuts, with some positive effects observed in all three. A double-blind, controlled, crossover trial [28] included 91 hypertensive and dyslipidemic patients, who received an individualized diet + 13 g granulated and defatted Brazil nuts (DBN) or individualized diet + placebo (IDP), found a 24% increase in the GPx3 activity (112.66 nmol/min/mL ± 40.09 nmol/min/mL to 128.32 nmol/min/mL ± 38.31 nmol/min/mL, *p* < 0.05) and 3.25% reduction in oxLDL (66.31 U/L ± 23.59 U/L to 60.68 U/L ± 20.88 U/L) in the DBN group at the end of 12 weeks. An inverse association between GPx3 and oxLDL was found, even after adjusting for sex, age, diabetes mellitus (DM) diagnosis, and body mass index (BMI) (β −0.298, *p* = 0.008). An increase in the GPx activity was also seen in the RCT [27] involving obese women who followed their usual diet and added one Brazil nut per day for two months when compared to the control (without any intervention) (*p* = 0.03). Obese adolescents, grouped to maintain their usual diets (control group) or a supplementary diet with 15 g–25 g of Brazil nuts per day (BNG group) for 16 weeks, showed a significant decrease in oxLDL in the BNG compared to the control group [BNG: 622.4 (457.2–665.0) to 514.9 (440.3–624.6); *vs.* control: 648.8 (515.9–737.9) to 646.9 (595–883.5); *p* = 0.02] [31].

In a study conducted with type-2 DM (T2DM) patients [75], duration of eight weeks and intervention with cashew nuts, 50 individuals were allocated to two groups: those with adjusted calories, receiving 10% of the total energy value (TEV) in cashew nuts (CNG); and those who followed their usual diets. At the end of the study, there was a greater increase in the activity of paraoxonase 1 (PON-1) in the CNG, but without significance. In another study [76], children and adolescents with primary hyperlipidemia (*n* = 60) were randomly assigned to three groups: the hazelnut group with skin, the hazelnut group without skin, and the control group. The amount of hazelnut varied from 15 g–30 g in both interventions and all followed dietary guidelines. There were no significant changes in oxLDL.

Two RCTs [32,77] were conducted in patients with metabolic syndrome (MS) with the intervention of 30 g/day of mixed nuts (in different compositions). In one [77], 60 volunteers were included and distributed into two groups (nuts or control); the mixed nuts were composed of 15 g walnuts, 7.5 g pine nuts, and 7.5 g roasted peanuts, all following nutritional guidelines. No changes in the oxidative stress markers were identified after six weeks of intervention. In the other study [32], 50 patients, receiving a healthy diet prescription, were followed up for 12 weeks, after being divided into two groups (nuts or control); the mixed nuts were composed of 15 g walnuts, 7.5 g almonds, and 7.5 g hazelnuts; after intervention, urinary levels of the biomarker 8-oxo-dG were significantly reduced (*p* < 0.001) in the walnut group.

A study [29] with 60 individuals with MS, lasting 24 weeks, divided patients into two groups: the pistachio group (PG) and the control group (CG). All participants followed a specific diet and exercise program, with unsalted pistachios making up 20% of the TEV for the PG. At the end of the study, the PG showed a significant improvement in TBARS compared to that in the control (*p* = 0.01). Another study [30] involving almonds, recruited 20 patients with T2DM and mild hyperlipidemia and divided them into two groups: the almond group (AG) and the control group (CG). The AG received a calorie-adjusted diet with 20% of the TEV from unsalted roasted almonds, while the CG received a diet from the National Cholesterol Education Program (NCEP). After four weeks, AG showed a significant reduction in carbonyl protein and oxLDL (28%, *p* = 0.0003 and 6.9%, *p* ≤ 0.05, respectively).

Studies previously described that evaluated the impact of different nuts on oxidative stress biomarkers in primary cardiovascular prevention are summarized in Table 4.

## 6. Nuts, Oxidative Stress Biomarkers, and Secondary Cardiovascular Prevention

Regarding the oxidative stress biomarkers already mentioned, although evaluated in clinical trials conducted with individuals in primary prevention, there is little data available in the literature on the effect of supplementation of nuts and associated antioxidants in patients with documented CVD. The evaluation of such aspects in this population is more complex since such patients are usually on medications that can modulate inflammation and oxidative stress.

For example, a crossover RCT [78] evaluated the effect of consumption of 85 g of almonds per day in 45 patients with documented CAD with 22 weeks of follow-up. It was observed in the group receiving intervention of almond nuts, that there was a 103% increase in the vitamin E intake, and a 10.3% increase in the serum levels of α-tocopherol and 17.5% in the urinary levels of NO compared to those observed in the control group (the NCEP diet), despite the short follow-up period and lack of statistical power to evaluate oxidative stress biomarkers.

A non-RCT [79] evaluated the intake of nuts in secondary prevention in the context of the MeDiet, with patients receiving the MeDiet (*n* = 21) or diet of a low-fat type (*n* = 19). Forty men aged 45 years–65 years, with a history of coronary events for more than four months and less than two years and stable at the time of the study, with LDL-c > 190 mg/dL, were followed for over three months. The recommendation for the MeDiet group was 10 g per day of nuts (Brazil nuts and/or almonds and/or walnuts). A reduction in the serum oxLDL in the low-fat diet group was observed, but the oxLDL:LDL-c ratio remained unchanged. This study also had a limited follow-up, beyond the inherent limitations of its design.

Additionally, there are studies in the literature that link oxidative stress and secondary cardiovascular prevention. For example, in the Cambridge Heart Antioxidant Study (CHAOS) study [80], nuts were not evaluated specifically, but there was supplementation with tocopherol—an important antioxidant present in nuts. This study randomized 2002 patients (mean age 61 years) with symptomatic CAD confirmed by angiography, to receive 400 IU–800 IU of daily vitamin E capsule, compared to that received by the placebo, followed up for approximately 510 days. There was a 47% reduction in the risk for non-fatal AMI, but no reduction in mortality.

In another study [81], the use of α-tocopherol (50 mg/day), β-carotene, or both, in 1862 male smokers with a history of AMI, with a follow-up of 5.3 years, was evaluated through an RCT. An increased relative risk of 1.58 (95% CI 1.05–2.40) in cardiovascular-related death in the group that received β-carotene in combination with α-tocopherol was observed, but there was no significant difference in the group receiving only supplementation with α-tocopherol as compared to that received by the placebo.

Aligned with these findings, the GISSI (*Gruppo Italiano per lo Studio della Sopravvivenza nell’Infarto miocardico*)-Prevenzione study [82], a RCT that evaluated the effects of PUFA omega-3 supplementation (1 g/day, *n* = 2836), vitamin E (300 mg/day, *n* = 2830), or both (*n* = 2830) in patients with a recent AMI (<3 months), found no beneficial effect of α-tocopherol supplementation on combined event of death, nonfatal MI, or stroke.

In order to clarify the conflicting results regarding vitamin E supplementation and secondary prevention, a Mendelian randomization study of two samples was conducted in 2019 to investigate the causal association between vitamin E and coronary heart disease (CHD). Three single nucleotide polymorphisms (SNP) were identified: rs964184, rs2108622, and rs11057830. The effect on CHD was assessed using vitamin E serum levels. Each 1 mg/L level increase was significantly associated with CAD among all participants [odds ratio (OR) 1.5, 95% CI 1:03–1:06] [83]. 

These results suggest that nuts may increase the plasma α-tocopherol and urinary NO levels even in polymedicated patients. However, the effects of vitamin E supplementation, not nut intake, on the outcomes in this population remain unclear. More robust data on the impact of nut consumption on oxidative stress markers and secondary cardiovascular prevention is needed.

## 7. Conclusions

An increase in oxidative stress favors atherosclerotic progression and is positively associated with ACS. The intake of antioxidant food sources and/or antioxidant supplements seem to contribute to the prevention and treatment of diseases through already-known mechanisms; nuts are a good example of such food sources due to their favorable palatability and dose-effect that allow their inclusion in the majority of diets.

The effects of nut consumption on oxidative stress parameters in primary and secondary cardiovascular prevention are promising, but remain unclear, especially in secondary prevention. The variability of the results can be attributed to factors such as: concentration of antioxidants in the food studied, the dosage used, duration of the intervention, and the characteristics of the study population. In this context, further studies on the impact of the consumption of different nuts on oxidative stress biomarkers, with clinical relevance in both primary and secondary cardiovascular prevention, will be needed.

## Figures and Tables

**Table 1:** Fatty acid composition of nuts and edible seeds.

Fatty Acids (g/100g of Lipids)	Nuts								Edible Seeds	
	Almond	Hazelnut	Macadamia	Walnut	Pecan	Pistachio	Brazil Nut	Cashew Nut	Peanut	Baru Almond
Saturated	9.19	8.25	16.09	9.81	7.33	14.6	25.35	21.12	14.81	18.77
Palmitic C16:0	7.43	5.57	8.88	7.15	5.09	12.61	15.11	10.7	7.2	7.39
Stearic C18:0	1.7	2.5	4.26	2.55	2.02	1.42	9.51	9.33	1.84	4.62
Arachid C20:0	0.06	0.14	2.95	0.07	0.06	0.35	0.25	0.63	1.19	1.10
MUFA	65.89	80.62	58.51	16.30	54.26	56.28	29.04	61.68	43.93	51.07
Palmitoleic C16:1	0.66	0.15	18.69	0.09	0.07	1.18	0.29	0.54	0.07	-
Oleic C18:1	65.89	80.52	58.51	16.14	53.65	55.98	28.75	61.15	42.48	48.37
Gadoleic C20:1	0.00	0.10	0.00	0.16	0.61	0.3	-	-	1.45	2.70
PUFA	23.95	10.57	4.39	72.79	37.95	27.11	45.61	17.19	37.81	32.35
Linoleic C18:2	23.85	10.43	1.81	60.23	37	26.55	45.43	16.88	37.52	30.13
Linolenic C 18:3	0.10	0.14	2.58	12.56	0.95	0.56	0.18	0.32	0.29	2.22

**Abbreviations:** MUFA: monounsaturated fatty acid; PUFA: polyunsaturated fatty acid. Adapted from Freitas and Naves, 2010 [52].

**Table 2:** Phytochemical content of nuts.

Phytochemical	Nuts							
	Almond	Hazelnut	Macadamia	Walnut	Pecan	Pistachio	Brazil Nut	Cashew Nut
Total phenolics (mg/100 g)	261	447	233	1602	1588	703	197	242
Proanthocyanidins (mg/100 g)	184.1	500.6	10	67.2	493.9	252.71	10	8.7
Flavonoids (mg/100 g)	25.01	13.21	137.9	0.54	2713.49	136.45	0.85	1.12
Phenolic acids and aldehydes (mg/100 g)	0.44	1.87	3.69	39.11	2052	1.27	11.35	-
Carotenoids (µg/100 g)	2	106	-	21	55	22832	-	31
Sterols (mg/100 g)	192.37	132.47	105.7	197.89	233.52	189.43	160.19	154

Adapted from Bolling et al., 2011 [46].

**Table 3:** Nutrient antioxidant concentration of different nuts and seeds per 100g.

	Cashew Nut [60]	Brazil Nut [57,60]	Almond [60]	Walnut [60,61]	Pecan [60]	Pistachio [60]	Macadamia [60]	Hazelnut [60,62]	Peanut [60,63]
Selenium (µg)	19.9	1917	4.1	4.9	3.8	7	3.6	2.4	9.3
Zinc (mg)	5.78	4.06	3.12	3.09	4.53	2.2	1.3	2.45	2.77
Vitamin A (μg)	0	0	0	1	3	26	0	1	0
Vitamin C (mg)	0.5	0.7	0	1.3	1.1	5.6	1.2	6.3	0
α-tocopherol (mg)	0.9	5.65	25.63	0.7	1.4	2.86	0.54	15.03	4.93
β-tocopherol (mg)	0.03	-	0.23	0.10	0.39	0	0	-	0.33
γ-tocopherol (mg)	5.31	116.2	0.64	22.65	24.44	1.67	0	1.36	10.4

**Table 4:** Randomized clinical trials on nuts and oxidative stress biomarkers in primary cardiovascular prevention.

Author; Location	RCT Design	Population (n: Intervention/Control)	Type of Nut	Duration	Intervention	Control	Biomarkers	Main Results	Conclusion
de Souza RGM et al. [26]; Brazil	Placebo-controlled	Women overweight and obese (n: 24/22)	Baru nut	8 weeks	Normocaloric and isocaloric diet-based Guideline + 20g Baru/day	Normocaloric and isocaloric diet-based Guideline	MDA, catalase, GPx, SOD	GPx intervention: +0.08; GPx control: -0.07. *p* <0.01	Baru nut supplementation increased GPx activity in women with excess of weight.
GBS Duarte et al. [27]; Brazil	Controlled	Women obese (n: 36/36)	Brazil nut	2 months	Usual diet + 1 unit Brazil nut/day	Usual diet	GPx1	GPx1 intervention: Δ8.5; GPx1 control: Δ 2.5. *p* =0.03	Brazil nut supplementation increased GPx1 activity in obese women.
Darvish Damavandi R et al. [75]; Iran	Controlled	Individuals with T2DM (n: 22/21)	Cashew nut	8 weeks	Adjusted calorie diet with 10% of cashew and reduced consumption of visible fat	Usual diet	TAC and PON-1	Intervention *vs* control: TAC *(p*= 0.34); PON-1 (*p*= 0.41).	Cashew nut did not improve TAC and PON-1 plasma activities in individuals with T2DM.
Guaraldi F et al. [76]; Italy	Controlled	Children and adolescents with hyperlipidemia (n: 42/18)	Hazelnut with or without skin	8 weeks	Nutritional recommendations based on CHILD-1 + hazelnut with or out skin 15 to 30g/day	Nutritional recommendations based on CHILD-1	oxLDL	oxLDL intervention *vs* control: *p*= 0.462.	Hazelnuts did not improve the oxLDL in children and adolescents with hyperlipidemia.
Huguenin GV et al. [28]; Brazil	Cross, double-blind, placebo-controlled	Individuals hypertension and dyslipidemia (n: 52/48)	Brazil nut partially defatted	12 weeks	Nutritional counseling for dyslipidemia and hypertension + Brazil nut 13g/day	Nutritional counseling for dyslipidemia and hypertension	GPx3, TAC, 8-epi PGF2α, oxLDL	GPx3 and oxLDL intervention: 128.32±38.31 60.68±20.88, respectively. GPx3 and oxLDL control: 115.06±38.09 and 63.76 ±23.03, respectively. *p* <0.05	Brazil nut intake increased GPx3 actitity and reduced oxLDL in individuals with hypertension and dyslipidemia.
Lee YJ et al. [77]; South Korea	Controlled	Individuals with MS (n: 30/30)	Mixed nuts (walnut, peanuts, and pine nuts)	6 weeks	Recommendations-based dietary guidelines + mix nuts 30g / day	Recommendations-based dietary guidelines	MDA, oxLDL	Serum and urine MDA and oxLDL intervention *vs* control: *p*= 0.445, *p*= 0.394, and *p*= 0.885, respectively.	Mixed nuts did not improve MDA and oxLDL in individuals with MS.
Bento AP et al. [74]; Brazil	Placebo-controlled, crossover	Mildly hypercholesterolemic (n: 20/20)	Baru nut	6 weeks	Usual diet + Baru 20g /day	Usual diet	TBARS, SOD, FRAP	TBARS, SOD, FRAP intervention *vs* control: *p*= 0.82, *p* =0.34, and *p* =0.33, respectively.	Baru nuts did not improve TBARS, SOD and FRAP in mildly hypercholesterolemic individuals.
Gulati S et al. [29]; India	Controlled	Asian Indian with MS (n: 33/35)	Pistachio nut unsalted	24 weeks	Guideline based on the standard diet with 20% of TEV in pistachio	Guideline based on the standard diet	TBARS	TBARS intervention: 2.4±1.3; TBARS control: 3.1±1.3. *p*= 0.01	Pistachio nut improved plasma TBARS in Asian Indian with MS.
Liu JF et al. [30]; Taiwan	Cross and controlled	Individuals with T2DM and mild hyperlipidemia (n: 20/10)	Almond nut	12 weeks	Diet for obtaining or maintaining weight with 20% of TEV in almond	Diet for obtaining or maintaining weight	carbonyl protein, oxLDL and MDA	Carbonyl protein intervention: 1.59±0.16; carbonyl protein control: 2.16 ±0.23 (p= 0.0003). oxLDL: reduced 6,9% with intervention as compared control. *p* ≤0.05	Almond reduced plasma carbonyl protein and oxLDL in individuals with T2DM and mild hyperlipidemia.
Maranhao PA et al. [31]; Brazil	Controlled	Obese adolescents (n: 8/9)	Brazil nuts	16 weeks	Usual diet + Brazil nut 15-25g/day	Usual diet	GPx3, oxLDL, 8-epi PGF2α	oxLDL: reduced in intervention group (622.4 to 514.9). *p*=0.02	Brazil nuts improved oxLDL in adolescents with obesity.
López-Uriarte P et al. [32]; Spain	Controlled	Individuals with MS (n: 25/25)	Mixed nuts (walnut, almond and hazelnut)	12 weeks	Nutritional guidelines + mix nuts 30g/day	Nutritional guidelines	plasma antioxidant capacity, oxLDL,8-oxo-dG, 8-isoprostane	Mean difference in final 8-oxo-dG (intervention *vs* control): Δ -2.42; *p* ≤0.001	Mixed nuts improved urinary 8-oxo-dG in individuals with MS.

**Abbreviations:** RCT: Randomized clinical trial; MDA: Malondialdehyde; GPx: Glutathione peroxidase; SOD: Superoxide dismutase; GPx1: Glutathione peroxidase 1; GPx3: Glutathione peroxidase 3; T2DM: Type-2 diabetes mellitus; TAC: Total antioxidant capacity; PON-1: Paraoxonase 1; 8-epi PGF2α: 8-epi-prostaglandin F2 alpha; oxLDL: Oxidized LDL cholesterol; MS: Metabolic syndrome; TBARS: Thiobarbituric acid reactive substances; FRAP: Ferric reducing antioxidant potential; 8-oxo-dG: 8-oxo-7,8-dihydro-2'-deoxyguanosine.
